# Prevalence-pattern of congenital and hereditary anomalies in Balochistan Province of Pakistan

**DOI:** 10.12669/pjms.40.9.9158

**Published:** 2024-10

**Authors:** Azmatullah, Muhammad Qasim Khan, Abdullah Jan, Junaid Mehmood, Sajid Malik

**Affiliations:** 1 Azmatullah, M.Phil Human Genetics Program, Department of Zoology, Faculty of Biological Sciences, Quaid-i-Azam University, Islamabad; 2 Muhammad Qasim Khan, M.Phil Human Genetics Program, Department of Zoology, Faculty of Biological Sciences, Quaid-i-Azam University, Islamabad; 3 Abdullah Jan, M.Phil Human Genetics Program, Department of Zoology, Faculty of Biological Sciences, Quaid-i-Azam University, Islamabad; 4 Junaid Mehmood, M.Phil Human Genetics Program, Department of Zoology, Faculty of Biological Sciences, Quaid-i-Azam University, Islamabad; 5 Sajid Malik, PhD Human Genetics Program, Department of Zoology, Faculty of Biological Sciences, Quaid-i-Azam University, Islamabad

**Keywords:** Birth defects, Epidemiology, Neurological disorders, Limb defects, Consanguinity

## Abstract

**Objectives:**

This study was aimed to report the prevalence-pattern of hereditary and congenital anomalies (CA) in general population of Balochistan province of Pakistan, and to elucidate the familial/sporadic presentations and parental consanguinity of CA.

**Methods:**

In a multi-center cross-sectional study, patients with CA were ascertained from various district hospitals and public places throughout Balochistan from 2019 to 2023. Online Mendelian Inheritance in Man (OMIM) and International Classification of Diseases (ICD-10) databases were utilized for uniformity in classification. Descriptive statistics was employed.

**Results:**

A cohort of 1185 independent patients diagnosed with CA was recruited and the index males were 71%. The CA were classified into nine major and 118 minor entities. In the major categories, neurological disorders had the highest prevalence (n=317; 27%), followed by limb defects (n=161; 14%), blood-heart defects (n=159; 13%), neuromuscular anomalies (n=156; 13%), sensorineural/ear defects (n=140; 12%), eye/visual impairments (n=90; 8%), musculoskeletal defects (n=83; 7%), ectodermal defects (n=31; 3%), and others (48; 4%). Sixty one percent CA were sporadic in nature and 39% were familial; and parental consanguinity was observed in 51% cases. Several rare CA were witnessed.

**Conclusions:**

High preponderance of sporadic presentations in neuromuscular anomalies and musculoskeletal defects and low incidence of parental consanguinity in ectodermal defects and musculoskeletal defects may depict a significant etiological role of non-genetic/environmental factors such as prenatal exposures and maternal conditions. In this context, it is important to increase health education, enhance antenatal and perinatal care, and strengthen the health-care system in Balochistan to reduce the burden of CA.

## INTRODUCTION

The hereditary and congenital anomalies (CA) are frequently observed in Pakistani communities. The burden of CA is generally higher in communities with low socioeconomic status, low literacy, high consanguinity, and poor antenatal care.^1^,^2^ Compared to other provinces of Pakistan, little is known about the prevalence-pattern of CA in Balochistan. In a recent study, Ijaz et al, recruited 105 consanguineous couples from Quetta and showed that 29 families had certain types of CA.^3^ Jesrani et al.^4^ reported congenital fetal anomalies diagnosed on antenatal ultrasound in a tertiary care hospital of Balochistan. Epidemiological accounts of thalassemia and Glucose-6-phasphate dehydrogenase deficiency in Quetta population were reported by Ahmed et al. and Raheem et al., respectively.^5^,^6^ Few studies have reported the molecular genetic diagnosis of rare CA like blindness, skeletal and neurological disorders.^7^-^9^ However, the overall burden of CA in Balochistan population has not been evaluated.

Balochistan province of Pakistan remains disadvantaged in healthcare sector. In comparison to the national average, many common health indicators are incredibly low. For instance, Balochistan had a neonatal mortality rate of 63 per 1000 live births, significantly higher than the national average of 42 per 1000.^10^ A recent study revealed that 46% of pregnant women in Balochistan had anemia.^11^ Compared to 23 out of 100 pregnant women in Punjab, Sindh, and Khyber Pakhtunkhwa, just 5 out of 100 women in Baluchistan used iron supplements for three months.^12^ Further, Balochistan had a skilled birth attendance rate of 38% compared to 75% in Sindh, 71% in Punjab, and 67% in Khyber Pakhtunkhwa.^13^

Only 18% of births in Balochistan occur in medical facilities, which makes it difficult to manage pregnancy risks. The prevalence of prenatal care in Pakistan is 86%, with Punjab leading the way with 92% coverage and Balochistan with 56%.^13^ Hence, Balochistan population is anticipated to carry heavy burden of CA which may have genetic, maternal, and environmental etiologies. The current clinico-epidemiological investigation is a preliminary step towards bridging this knowledge gap.

## METHOD

A multi-center study was conducted in Balochistan, Pakistan from 2019-2023 and patients/families with CA were recruited from Civil Hospital Quetta and District Headquarter Hospitals in Pishin, Zhob, Loralai and Ziarat. In order to have representation of all socio-demographic strata and age segments, patients were also recruited from general populations through mixed method sampling including door-to-door visits, whereas snowball sampling was employed in the rural areas. Individual having congenital and hereditary anomalies with an explicit phenotypic manifestation were included. Patients/families were recruited irrespective of gender, ethnicity, or the anomalies they displayed. Individuals with late onset noncommunicable diseases and with clear poliomyelitis presentation were not considered. Individuals with psychiatric or behavioral disorders as well as anomalies with accidental, trauma or infectious origin, were excluded.

### Ethical Consideration:

The Ethical Review Committee at Quaid-i-Azam University, Islamabad gave the formal approval of the study (DAS-15-, June 3, 2015). All participants provided informed consent, in line with the principles outlined in the Helsinki-II declaration. The study followed the reporting guidelines specified by the Strengthening the Reporting of Observational Studies in Epidemiology (STROBE) statement for cross-sectional studies.^14^

### Classification and statistical analyses:

The CA were diagnosed with the help of qualified medical professionals at the respective district hospitals. Pre-diagnosed cases from rehabilitation and disability centers were also included. CA were classified into broader categories: neurological disorders, neuromuscular anomalies, musculoskeletal defects, eye/visual impairments, sensorineural/ear defects, limb defects, and blood-heart disorders. Classification of anomalies was further refined with the help of OMIM (https://www.omim.org/) and ICD-10 databases (https://icd.who.int/browse10/2019/en).

Detailed pedigrees were constructed for each individual but only the index person from a particular family was included in the analyses. Descriptive summaries were generated, and Chi-square and Fisher-exact tests were employed to check the significance of distribution. For the CA, proportions and corresponding 95% confidence intervals (CI) were calculated.

## RESULTS

### Sample characteristics:

A total of 1185 index cases, 839 (71%) males and 346 (29%) females, were included in the study (Table-I). There was representation of individuals from all districts of Balochistan and the individuals belonging to Loralai and Quetta districts were 40% and 20%, respectively. Majority of the individuals originated from rural areas (57%), spoke Pashto (78%), and belonged to poor and low socioeconomic quartiles (56%). The individuals between >9-19 years of age had the highest representation (39%).

**Table-I:** Demographic distribution of index subjects.

Variables	Male, No. (%)	Female, No. (%)	Total, No. (%)
***Districts***			
Loralai	320 (67)	158 (33)	478 (40)
Quetta	161 (69)	72 (31)	233 (20)
Zhob	156 (75)	52 (25)	208 (18)
Ziarat	83 (78)	23 (22)	106 (9)
Pishin	34 (71)	14 (29)	48 (4)
Others	85 (76)	27 (24)	112 (9)
Total	839 (71)	346 (29)	1185 (100)
Rural/urban origin*			
Rural	387 (58)	284 (42)	671 (57)
Urban	452 (88)	62 (12)	514 (43)
***Mother tongue***			
Pashto	656 (71)	268 (29)	924 (78)
Brohvi	55 (69)	25 (31)	80 (7)
Balochi	58 (73)	21 (27)	79 (7)
Saraiki	21 (70)	9 (30)	30 (3)
Others	49 (68)	23 (32)	72 (6)
Economic quartile*			
Poor	237 (20)	96 (8)	333 (28)
Low	215 (18)	115 (10)	330 (28)
Low-mid	344 (29)	125 (11)	469 (40)
High-mid	43 (4)	10 (1)	53 (4)
Age categories (years)*			
Up to 5	143 (65)	76 (35)	219 (18)
>5-9	134 (63)	78 (37)	212 (18)
>9-19	325 (71)	135 (29)	460 (39)
>19-29	148 (80)	36 (20)	184 (16)
>29	89 (81)	21 (19)	110 (9)

* differences in the distribution were statistically significant.

### Major congenital anomalies:

The CA observed in this cohort were categorized into 9 major and 122 minor categories (Table-II, III). Among the major categories, neurological disorders had the highest representation (n=317; 27%), followed by limb defects (n=161; 14%), blood-heart disorders (n=159; 13%), neuromuscular disorders (n=156; 13%), sensorineural/ear defects (n=140; 12%), eye/visual impairments (n=90; 8%), musculoskeletal defects (n=83; 7%), ectodermal anomalies (n=31; 3%), and others (n=48; 4%) (Table-II).

**Table-II:** Index cases in major categories of CA, proportions and 95% CI.

Major category	Index cases			Proportion	95% CI

	Male, No. (%)	Female, No. (%)	Total		
Neurological disorders	236 (74)	81 (26)	317	0.268	0.288-0.348
Limb defects	116 (72)	45 (28)	161	0.136	0.260-0.293
Blood-heart disorders	109 (69)	50 (31)	159	0.134	0.258-0.291
Neuromuscular anomalies	110 (71)	46 (29)	156	0.132	0.255-0.288
Sensorineural/ear defects	97 (69)	43 (31)	140	0.118	0.232-0.270
Eye/visual impairments	59 (66)	31 (34)	90	0.076	0.191-0.230
Musculoskeletal defects	49 (59)	34 (41)	83	0.070	0.166-0.199
Ectodermal defects	19 (61)	12 (39)	31	0.026	0.114-0.228
Others	44 (92)	4 (8)	48	0.041	0.137-0.183
Total	839 (71)	346 (29)	1185	1.000	

Chi2=12.24, P=0.141.

**Table-III:** Major and minor categories of congenital anomalies.

Major/minor categories	Frequency	Proportion	95% CI	ICD-10	OMIM
**Neurological disorders**	**317**	**0.268**	**0.242-0.293**		
Intellectual disability (undefined)	91	0.077	0.062-0.092	F03	300243
Intellectual disability, moderate	60	0.051	0.038-0.063	F71	
Intellectual disability, mild	53	0.045	0.033-0.056	F70	249500
Down syndrome	23	0.019	0.012-0.027	Q90	190685
Intellectual disability, severe	22	0.019	0.011-0.026	F72, F73	611091
Epilepsy	19	0.016	0.009-0.023	G40	117100
Spina bifida	10	0.008	0.003-0.014	Q05	182940
Intellectual disability, profound	8	0.007	0.002-0.011	F73	
Epilepsy, juvenile type	7	0.006	0.002-0.010		604827
Microcephaly	6	0.005	0.001-0.009	Q02	251200
Hydrocephaly	3	0.003	0.000-0.005	G91.9	236600
Macrocephaly	3	0.003	0.000-0.005	Q75.3	153470
Encephalocele	2	0.002	-0.001-0.004	Q01	
Epilepsy: status epilepticus	2	0.002	-0.001-0.004	G41	
Metachromatic leukodystrophy	2	0.002	-0.001-0.004	E75.2	250100
Myelomeningocele	2	0.002	-0.001-0.004	Q05	
Agenesis of corpus collosum	1	0.001	-0.001-0.002	Q04.0	218000
Cerebral atrophy	1	0.001	-0.001-0.002	G31.9	619244
Dandy-Walker syndrome	1	0.001	-0.001-0.002	Q03.1	220200
Developmental delay	1	0.001	-0.001-0.002	Z13.42	618330
**Limb defects**	**161**	**0.136**	**0.116-0.155**		
Talipes	58	0.049	0.037-0.061	Q66.0	119800
Polydactyly, postaxial	21	0.018	0.010-0.025	Q69.0	174200
Congenital amputations	20	0.017	0.010-0.024	Q73.0, Q72.0	217100
Polydactyly types	16	0.014	0.007-0.020	Q69.9	174200, 174400
Polydactyly, preaxial	16	0.014	0.007-0.020	Q69.1	174400
Syndactyly types	12	0.010	0.004-0.016	Q70	186000
Zygodactyly	4	0.003	0.000-0.007		609815
Brachydactyly	4	0.003	0.000-0.007	Q68.81	113000
Congenital dislocation of knee	3	0.003	0.000-0.005	Q68.2	
Camptodactyly	1	0.001	-0.001-0.002	Q74.0	114200
Clinodactyly	1	0.001	-0.001-0.002	Q74.0	148520
Humeral hypoplasia	1	0.001	-0.001-0.002	Q71.8	
Hypertrophy of foot	1	0.001	-0.001-0.002	Q74.2	
Macrodactyly	1	0.001	-0.001-0.002	Q74.2	155500
Split foot/oligodactyly	1	0.001	-0.001-0.002	Q72.7	183600
Split-hand and syndactyly	1	0.001	-0.001-0.002	Q71.6	183600
**Blood-Cardiac disorders**	**159**	**0.134**	**0.115-0.154**		
Thalassemia major	118	0.100	0.083-0.117	D56	613985
Atrial septal defect	13	0.011	0.005-0.017	Q21.1	108800
Thalassemia minor	8	0.007	0.002-0.011	D56.3	
Atrio-ventricular septal defect	5	0.004	0.001-0.008	Q21.2	606215
Hemophilia	4	0.003	0.000-0.007	D66	306700
Sickle cell anemia	4	0.003	0.000-0.007	D57.1	603903
Anemia	2	0.002	-0.001-0.004	D64.9	105600
Cardiomyopathy	1	0.001	-0.001-0.002	I42	115200
Leukemia	1	0.001	-0.001-0.002	C95.9	613065
Thrombophilia	1	0.001	-0.001-0.002	D68.5	188055
Truncus arteriosus	1	0.001	-0.001-0.002	Q20.0	217095
Ventricular septal defect	1	0.001	-0.001-0.002	Q21.0	614429
**Neuromuscular anomalies**	**156**	**0.132**	**0.112-0.151**		
Cerebral palsy types	127	0.107	0.090-0.125	G80	605388
Muscle hypotonia	19	0.016	0.009-0.023	P94.2	
Muscular dystrophy (Duchenne)	6	0.005	0.001-0.009	G71.2	310200
Muscular dystrophy (Becker)	3	0.003	0.000-0.005	G71.2	300376
Muscular dystrophy	1	0.001	-0.001-0.002	G71.2	253700
**Sensorineural/ear defects**	**140**	**0.118**	**0.100-0.137**		
Deaf and mute	80	0.068	0.053-0.082	H91.3	304500
Stuttering	32	0.027	0.018-0.036	F98.5	184450
Mute only	15	0.013	0.006-0.019	R47.0	
Deaf only	6	0.005	0.001-0.009	H90	601072
Microtia	5	0.004	0.001-0.008	Q17.2	600674
Pinna deformed	2	0.002	-0.001-0.004	Q17.3	
**Eye/visual impairments**	**90**	**0.076**	**0.061-0.091**		
Blindness (undefined)	26	0.022	0.014-0.030	H54	216900
Strabismus	18	0.015	0.008-0.022	H50.9	185100
Congenital conjunctivitis	9	0.008	0.003-0.013	H10.9	
High myopia	8	0.007	0.002-0.011	H52.10	601075
Retinitis pigmentosa	8	0.007	0.002-0.011	H35.5	603937
Night blindness	5	0.004	0.001-0.008	H53.60	310500
Cataract	3	0.003	0.000-0.005	Q12.0	115700
Color blindness	3	0.003	0.000-0.005	H53.5	303800
Day Blindness	3	0.003	0.000-0.005	H53.1	
Heterochromia	3	0.003	0.000-0.005	Q13.2	142500
Anophthalmia	2	0.002	-0.001-0.004	Q11.2	251600
Blepharospasm	1	0.001	-0.001-0.002	G24.5	606798
Ptosis	1	0.001	-0.001-0.002	Q10.0	300245
**Musculoskeletal defects**	**83**	**0.070**	**0.056-0.085**		
Developmental dysplasia of hip	23	0.019	0.012-0.027	Q65.8	142700
Dwarfism (undefined)	15	0.013	0.006-0.019	E34.3	100800
Anisomelia	12	0.010	0.004-0.016	Q72.9	
Arthrogryposis	8	0.007	0.002-0.011	Q74.3	108120
Achondroplasia	5	0.004	0.001-0.008	Q77.4	100800
Kyphoscoliosis	4	0.003	0.000-0.007	M40	610170
Skeletal dysplasia	4	0.003	0.000-0.007	Q79	618870
Mucopolysaccharidosis	2	0.002	-0.001-0.004	E76.3	252800
Vitamin-D resistant rickets	2	0.002	-0.001-0.004	E83.3	277440
DuPan syndrome	1	0.001	-0.001-0.002		228900
Ellis-van Creveld syndrome	1	0.001	-0.001-0.002	Q77.6	225500
Exostosis	1	0.001	-0.001-0.002	Q78.6	133700
Genu valgum	1	0.001	-0.001-0.002	M21.06	137370
Klippel-Feil syndrome	1	0.001	-0.001-0.002	Q76.1	118100
Osteogenesis imperfecta	1	0.001	-0.001-0.002	Q78.0	166200
Pierre-Robinsons syndrome	1	0.001	-0.001-0.002	Q87.0	
Tall stature-obesity syndrome	1	0.001	-0.001-0.002		
**Ectodermal defects**	**31**	**0.026**	**0.017-0.035**		
Xeroderma pigmentosum	5	0.004	0.001-0.008	Q82.1	278730
Vitiligo	4	0.003	0.000-0.007	L80	606579
Albinism	3	0.003	0.000-0.005	E70.3	203100
Epidermolysis bullosa	3	0.003	0.000-0.005	Q81	226650
Ichthyosis	3	0.003	0.000-0.005	Q80	242300
Neurofibromatosis	3	0.003	0.000-0.005	Q85.0	162200
Darrier disease (keratosis follicularis)	2	0.002	-0.001-0.004	Q82.8	308800
Dyskeratosis congenita	2	0.002	-0.001-0.004		615190
Chronic bullous disease of childhood	1	0.001	-0.001-0.002	L12.2	
Hyperelastic skin (Cutis laxa)	1	0.001	-0.001-0.002	Q82.8	219200
Hypotrichosis	1	0.001	-0.001-0.002	Q84.0	605389
Lipoid proteinosis	1	0.001	-0.001-0.002	E78.8	247100
Nail atrophy	1	0.001	-0.001-0.002	L60.3	161050
Psoriasis	1	0.001	-0.001-0.002	L40	177900
**Others**	**48**	**0.041**	**0.029-0.052**		
Cleft lip-palate	13	0.011	0.005-0.017	Q37	119530
Bardet-Biedl syndrome	6	0.005	0.001-0.009	Q87.89	209900
Diabetes mellitus	6	0.005	0.001-0.009	P70.2	222100
Anosmia	4	0.003	0.000-0.007	R43.0	
Asthma/severe allergic response	3	0.003	0.000-0.005	J45	600807
Cleft lip only	2	0.002	-0.001-0.004	Q36	600625
Hypospadias	2	0.002	-0.001-0.004	Q54.1	
Citrullinemia	1	0.001	-0.001-0.002	E72.2	215700
Congenital adrenal hyperplasia	1	0.001	-0.001-0.002	E25.0	201910
Congenital erythropoietic porphyria	1	0.001	-0.001-0.002	E80.0	263700
Dysmorphic/asymmetric face	1	0.001	-0.001-0.002	Q67.0	
Enteric malrotation, coeliac disease	1	0.001	-0.001-0.002	Q43.9	212750
Hirschsprung’s disease	1	0.001	-0.001-0.002	Q43.1	142623
Holt-Oram syndrome	1	0.001	-0.001-0.002	Q87.2	142900
Hypercholesterolemia	1	0.001	-0.001-0.002	E78.0	603813
Nephrotic syndrome	1	0.001	-0.001-0.002	N04	600995
Polycystic kidney disease	1	0.001	-0.001-0.002	Q61.1	263200
Pulmonary hypertension	1	0.001	-0.001-0.002	127.O	178600
Wilson’s disease	1	0.001	-0.001-0.002	E83.01	277900

### Spectrum of anomalies:

There were 317 individuals involving neurological disorders. Among these, intellectual disabilities (ID), epilepsy, and Down syndrome were most prominent. Collectively, ID were 263 (including Down syndrome and microcephaly). Among the limb defects, there were 13 distinct entities. The most common malformation was clubfoot (n=58), followed by polydactyly (all types, n=53), amputation/deficiencies (n=20), and syndactyly (including zygodactyly, n=16). Among the blood-heart disorders, the individuals with thalassemia were highly represented (including minor type; n=126), followed by atrial septal defect (n=13). Thalassemia major alone involved 118 individuals.

Among the neuromuscular disorders, cerebral palsy (CP) was most prominent (n=127), followed by muscle hypotonia (n=19). In cerebral palsy, spastic and athetoid types were most common comprising 55% and 30% cases, respectively. CP was the second most prevalent entity after ID. Among the CP types, spastic cases were n=70, athetoid n=38 and ataxic n=10.

### Familial-sporadic nature:

The anomalies with sporadic appearance were more common compared to the familial (61% vs. 39%, respectively; Table-IV), and the differences among the major anomalies were statistically significant. There was highest representation of sporadic cases in neuromuscular anomalies, followed by musculoskeletal defects, limb defects and neurological disorders (75%, 71%, 66% and 64%, respectively). On the other hand, familial occurrence was more frequent in ectodermal defects and eye/visual defects (74% and 59%, respectively).

**Table-IV:** Major categories of CA, parental consanguinity, familial/sporadic nature, and total affected family members.

Major category	Index cases	Parental consanguinity, No. (%)		Familial/sporadic nature, No. (%)		Total affected in all families, No. (%)		

		Present	Absent	Familial	Sporadic	Male	Female	Total
Neurological disorders	317	164 (52)	153 (48)	113 (36)	204 (64)	386 (70)	164 (30)	550
Limb defects	161	70 (43)	91 (57)	54 (34)	107 (66)	184 (65)	98 (35)	282
Blood-heart disorders	159	70 (44)	89 (56)	77 (48)	82 (52)	212 (69)	95 (31)	307
Neuromuscular anomalies	156	78 (50)	78 (50)	39 (25)	117 (75)	147 (67)	72 (33)	219
Sensorineural/ear defects	140	76 (54)	64 (46)	56 (40)	84 (60)	228 (68)	108 (32)	336
Eye/visual impairments	90	48 (53)	42 (47)	53 (59)	37 (41)	139 (68)	64 (32)	203
Musculoskeletal defects	83	51 (61)	32 (39)	24 (29)	59 (71)	78 (58)	56 (42)	134
Ectodermal defects	31	23 (74)	8 (26)	23 (74)	8 (26)	81 (61)	52 (39)	133
Others	48	27 (56)	21 (44)	21 (44)	27 (56)	76 (70)	33 (30)	109
Total	1,185	607 (51)	578 (49)	460 (39)	725 (61)	1531 (67)	742 (33)	2273

Chi2=12.24, P=0.141; Chi2=57.6; P<0.0001

Among all families, there were a total of 2273 affected individuals, comprising 1531 (67%) males and 742 (33%) females (P=0.0005). The highest proportion of affected females was witnessed in musculoskeletal defects and ectodermal defects (42% and 39%, respectively).

### Parental consanguinity:

Parental consanguinity was present in 51% of cases and the differences among the major categories were statistically not significant (Table-IV). Highest rate of parental consanguinity was observed in ectodermal defects (74%) followed by musculoskeletal defects (61%), whereas limb defects had the lowest consanguinity rate (42%).

### Geographic and ethnic differences in CA:

The differences in the distribution of major categories of CA among the districts were statistically significant (p<0.0001) (Table-V). Detailed analyses showed that neurological disorders were more prevalent in individuals from Quetta (39%), blood-heart disorders and neuromuscular defects in Lorali (21% and 19%, respectively), sensorineural defects in Ziarat (20%), eye/visual impairments in Zhob (14%), and musculoskeletal defects were more common in individuals coming from Pishin (15%). Further, with respect to mother tongue, the differences in the distribution of major categories of CA were statistically significant (p<0.0001) (Table-V). These analyses revealed that neurological disorders were more prevalent in individuals speaking Brohvi, limb defects in Balochi speaking, blood-heart disorders in Pashto speaking, neuromuscular defects in Brohvi speaking, and eye/visual impairments were more prevalent in Balochi speaking individuals.

**Table-V:** Geographic and ethnic differentials distribution of major categories of CA.

Variable	Neurological disorders	Limb defects	Blood-heart disorders	Neuro-muscular anomalies	Sensori-neural/ ear defects	Eye/visual impairments	Musculo-skeletal defects	Ectodermal defects	Others	Total
Districts*										
Loralai	101 (21)	77 (16)	98 (21)	89 (19)	55 (12)	24 (5)	24 (5)	0	10 (2)	478
Quetta	92 (39)	27 (12)	14 (6)	31 (13)	9 (4)	15 (6)	22 (9)	13 (6)	10 (4)	233
Zhob	70 (34)	11 (5)	21 (10)	10 (5)	40 (19)	29 (14)	17 (8)	2 (1)	8 (4)	208
Ziarat	24 (23)	13 (12)	16 (15)	14 (13)	21 (20)	4 (4)	3 (3)	2 (2)	9 (8)	106
Pishin	12 (25)	8 (17)	2 (4)	5 (10)	0	3 (6)	7 (15)	4 (8)	7 (15)	48
Others	18 (16)	25 (22)	8 (7)	7 (6)	15 (13)	15 (13)	10 (9)	10 (9)	4 (4)	112
Total	317 (27)	161 (14)	159 (13)	156 (13)	140 (12)	90 (8)	83 (7)	31 (3)	48 (4)	1,185
Mother tongue**										
Pashto	238 (26)	113 (12)	139 (15)	122 (13)	119 (13)	66 (7)	70 (8)	16 (2)	41 (4)	924
Brohvi	23 (28)	11 (13)	6 (7)	16 (20)	9 (11)	7 (9)	4 (5)	3 (4)	1 (1)	80
Balochi	14 (18)	23 (29)	5 (6)	4 (5)	8 (10)	9 (11)	4 (5)	8 (10)	4 (5)	79
Saraiki	7 (23)	5 (17)	3 (10)	7 (23)	3 (10)	1 (3)	2 (7)	2 (7)	0	30
Others	35 (49)	9 (13)	6 (8)	7 (10)	1 (1)	7 (10)	3 (4)	2 (3)	2 (3)	72

* Chi2=248.1, P<0.0001;

** Chi2=87.40, P<0.0001.

## DISCUSSION

This study presents detailed prevalence-pattern and clinical aspects of CA recruited from Balochistan province of Pakistan. This is the first study of such kind which involves a large sample size ascertained through a multi-center study. Parental consanguinity has been shown to be one of the detrimental factors in CA.^1^,^3^ Curiously however, parental consanguinity in our cohort was 51% which is significantly low compared to the Kashmir and Punjabi communities of Pakistan.^15^-^17^ Previously, Mian and Mushtaq showed that the rate of consanguinity in population of Quetta was 31%.^18^ In a recent study in Quetta, Ijaz et al, enrolled 105 couples with consanguineous marriages and observed that child mortality and CA were high among the couples with first cousin marriages.^3^ The authors showed that the common CA included mental retardation (24%), diabetes (10%), deafness (7%), and thalassemia (6.8%).

Neurological disorders had the highest representation in this study. This is similar to other studies carried out in Khyber Pakhtunkhwa, Pakistan.^1^,^19^,^20^ Pakistan bares a substantial burden of ID as compared to other developing countries. Several factors contribute to the high prevalence of ID in developing nations, which include maternal age, education, socioeconomic status, rural upbringing, suboptimal antenatal care, malnutrition, and infections.^21^,^22^

In the present cohort, cerebral palsy (CP) was the most common anomaly in neuromuscular disorders (n=127). CP is a common developmental disorder prevalent in families with socioeconomically deprived strata. In Pakistan, CP is one of the common causes of disability and morbidity. Among the common risk factors of CP are maternal malnutrition, premature birth, poor antenatal care, birth asphyxia and infections of nervous system are the major causes of CP.^23^ In the rural areas of Pakistan in general and in Balochistan in particular, there is high ratio of maternal malnutrition and anemia, and majority of the deliveries take place at home. Hence, an overwhelmingly high ratio of sporadic cases was observed with CP indicating low contribution of genetic factors. In these data, spastic CP and athetoid CP were most common comprising 55% and 30% of the CP cases, respectively. Nonetheless, among spastic CP, quadriplegia is more prevalent followed by diplegia and hemiplegia.

In present cohort, in the category of eye/visual impairments, blindness types and strabismus were observed in 26 and 18 individuals, respectively. Lakho et al^24^ carried out a study on ocular problems prevalent among the school children in Lasbela district of Balochistan. It was observed that 23% children had ocular problems, and conjunctivitis was the most common condition (11%), followed by vitamin-A deficiency disorders (3.3%), and refractive errors (3%). Notably, the ocular problems were more prevalent among children in *madrasa* (34%) compared to conventional schools (21%), with significant differences seen in conjunctivitis (17% in children from *madaris* vs. 10% in schools).

Here, the sporadic cases were almost twice as common as compared to familial cases (65% vs. 35%), which is similar to a study carried out in other regions of Pakistan.^17^,^19^ Concordantly, Naeem et al.^20^ also witnessed high incidence of sporadic cases among the cohort of CA ascertained from north-western territories of Pakistan. The high prevalence of sporadic cases among the limb and neurological disorders, coupled with lower levels of parental consanguinity, may suggest a likely contribution of nongenetic factors in the etiology of these anomalies.

### Limitations:

This study has several limitations. It does not provide the true prevalence of CA, rather the anomalies ascertained based on convenience sampling were reported. Hence, the rates presented here may by under- or over-estimated. The diagnosis of CA is mainly based on the phenotypic presentation and is not supported by molecular genetic methods. The environmental and risk factors which may be involved in the etiology of the CA were not explored in this study.

## CONCLUSION

Our research indicates that non-genetic and environmental factors may play a significant role in the development of sizable number of CA, particularly those affecting the neuromuscular and musculoskeletal systems. Given this context in resource deficient region like Balochistan, it is crucial to take several measures to address and reduce the burden of CA. These measures include: 1) increasing health education; 2) enhancing antenatal and perinatal care; 3) provision of premarital counseling and genetic testing; 4) implementing genetic screening programs particularly for common anomalies like thalassemia and metabolic disorders; and strengthening the health-care system.

## Authors’ Contributions:

**SM:** Conceived, designed and supervised the study.

**Azm**, **MQK**, **AJ** and **JM:** Data collection and statistical analysis. **SM:** Responsible and accountable for the accuracy and integrity of data.

## References

[1] Shaheen F, Humayoon QS, Malik S, Mumtaz S (2023). Clinical and genetic attributes of congenital anomalies ascertained at a tertiary care hospital in Rawalpindi, Pakistan. Pak J Med Sci.

[2] Raza MZ, Sheikh A, Ahmed SS, Ali S, Naqvi SM (2012). Risk factors associated with birth defects at a tertiary care center in Pakistan. Italian J Pediatr.

[3] Ijaz A, Ali MM, Kausar S, Saif K, Hussain MM, Tawakal RM (2022). Pattern and prevalence of congenital malformation and genetic disorders among offspring of consanguineous parents in Quetta. Pak-Euro J Med Life Sci.

[4] Jesrani A, Gul P, Jogezai S, Gul P, Naheed F, Jamal A (2019). Analysis of frequency of congenital fetal anomalies diagnosed on antenatal ultrasound in a tertiary care hospital of Balochistan. Int J Reprod Contracep Obstet Gynecolo.

[5] Ahmed S, Ayub M, Naeem M, Nazir FH, Hussain A, Ghilzai D (2021). Thalassemia patients from Baluchistan in Pakistan are infected with multiple hepatitis B or C virus strains. Am J Trop Med Hygiene.

[6] Raheem A, Sajid M, Fatima N (2019). Prevalence of G6PD deficiency in newborns of local community of Quetta, Pakistan. Wd J Pharma Med Res.

[7] Ijaz A, Basit S, Gul A, Batool L, Hussain A, Afzal S (2019). XPC gene mutations in families with xeroderma pigmentosum from Pakistan;prevalent founder effect. Cong Anom.

[8] Sajjad N, Goebel I, Kakar N, Cheema AM, Kubisch C, Ahmad J (2008). A novel HSF4 gene mutation (p. R405X) causing autosomal recessive congenital cataracts in a large consanguineous family from Pakistan. BMC Med Genet.

[9] Muhammad N, Hussain SI, Rehman ZU, Khan SA, Jan S, Khan N (2023). Autosomal recessive variants c.953A>C and c.97-1G>C in NSUN2 causing intellectual disability:a molecular dynamics simulation study of loss-of-function mechanisms. Frontiers Neurol.

[10] Mahmood A, Sultan M (2006). National Institute of Population Studies (NIPS) (Pakistan), and Macro International Inc. Pak Demographic Health Survey.

[11] Ashraf B (2023). Frequency of Anemia and Associated Risk Factors among Pregnant Women;A Study From The Remote Outskirts of Quetta, Balochistan. J Soc Obstet Gynaecol Pak.

[12] Japan International Cooperation Agency;International Techno Center Co. Ltd (2018). Data collection survey on health facilities and equipment in the Islamic Republic of Pakistan:Final report.

[13] Dawood Z, Majeed N (2022). Assessing neo-natal mortality trends in Pakistan:an insight using equity lens. Arch Public Health.

[14] Elm EV, Altman DG, Egger M, Pocock SJ, Gotzsche PC, Vandenbroucke JP (2007). Strobe initiative-the strengthening the reporting of observational studies in epidemiology (STROBE) statement:Guidelines for reporting observational studies. Epidemiology-Baltimore.

[15] Jabeen N, Malik S (2014). Consanguinity and its sociodemographic differentials in Bhimber district, Azad Jammu and Kashmir, Pakistan. J Health Pop Nutrit.

[16] Hina S, Malik S (2015). Pattern of consanguinity and inbreeding coefficient in Sargodha district, Punjab, Pakistan. J Biosoc Sci.

[17] Nawaz A, Zaman M, Malik S (2021). Consanguinity, inbreeding coefficient, fertility and birth-outcome in population of Okara district, Pakistan. Pak J Med Sci.

[18] Mian A, Mushtaq R (1994). Consanguinity in population of Quetta (Pakistan):a preliminary study. J Hum Ecol.

[19] Bibi A, Naqvi SF, Syed A, Zainab S, Sohail K, Malik S (2022). Burden of Congenital and Hereditary Anomalies in Hazara Population of Khyber Pakhtunkhwa, Pakistan. Pak J Med Sci.

[20] Naeem M, Ahmad B, Malik S (2022). Burden of congenital and hereditary anomalies in the war-affected territory at Pakistan–Afghanistan border. Asian Biomed.

[21] Ibrahim SH, Rizvi A, Ahmed AM, Bhutta ZA (2022). Association of risk factors for early childhood disability in rural Pakistan. Asia Pacific J Pub Health.

[22] Naz S, Ibrahim N, Sharif S, Bashir N, Sajjad E, Asghar I (2021). Prevalence and association of different levels of intellectual disability with prenatal, perinatal, neonatal and postnatal factors:prevalence and association of levels of ID. Proc Pak Acad Sci.

[23] Mughal M, Rizvi SM, Malik S (2022). Cerebral Palsy in Pakistan:A Review of Study Approaches, Status of Research and Trends. Pak Pediatr J.

[24] Lakho KA, Jadoon MZ, Mahar PS (2012). Pattern of Ocular Problems in School going Children of District Lasbela, Balochistan. Pak J Ophthalmol.

